# Hydroxyapatite-Integrated, Heparin- and Glycerol-Functionalized Chitosan-Based Injectable Hydrogels with Improved Mechanical and Proangiogenic Performance

**DOI:** 10.3390/ijms23105370

**Published:** 2022-05-11

**Authors:** Fatma Z. Kocak, Muhammad Yar, Ihtesham U. Rehman

**Affiliations:** 1 Engineering-Architecture Faculty, Metallurgy and Material Engineering, Nevsehir Haci Bektas Veli University, Nevsehir 50300, Turkey; fzkocak@nevsehir.edu.tr; 2 Engineering Department, Lancaster University, Lancaster LA1 4YW, UK; 3 Interdisciplinary Research Centre in Biomedical Materials (IRCBM), COMSATS University Islamabad, Lahore Campus, Lahore 54000, Pakistan; drmyar@cuilahore.edu.pk

**Keywords:** angiogenesis, bone tissue regeneration, glycerol, injectable hydrogels, minimal invasive surgery, pH sensitive, pro-angiogenic, thermosensitive, vascularisation

## Abstract

The investigation of natural bioactive injectable composites to induce angiogenesis during bone regeneration has been a part of recent minimally invasive regenerative medicine strategies. Our previous study involved the development of in situ-forming injectable composite hydrogels (Chitosan/Hydroxyapatite/Heparin) for bone regeneration. These hydrogels offered facile rheology, injectability, and gelation at 37 °C, as well as promising pro-angiogenic abilities. In the current study, these hydrogels were modified using glycerol as an additive and a pre-sterile production strategy to enhance their mechanical strength. These modifications allowed a further pH increment during neutralisation with maintained solution homogeneity. The synergetic effect of the pH increment and further hydrogen bonding due to the added glycerol improved the strength of the hydrogels substantially. SEM analyses showed highly cross-linked hydrogels (from high-pH solutions) with a hierarchical interlocking pore morphology. Hydrogel solutions showed more elastic flow properties and incipient gelation times decreased to just 2 to 3 min at 37 °C. Toluidine blue assay and SEM analyses showed that heparin formed a coating at the top layer of the hydrogels which contributed anionic bioactive surface features. The chick chorioallantoic membrane (CAM) assay confirmed significant enhancement of angiogenesis with chitosan-matrixed hydrogels comprising hydroxyapatite and small quantities of heparin (33 µg/mL) compared to basic chitosan hydrogels.

## 1. Introduction

Composite biomaterials based on natural biodegradable polymers enriched with inorganic minerals and other bioactive agents have recently attracted the attention of those working on guided tissue regeneration and drug delivery applications. Physically cross-linked hydrogels are biomimetic and biocompatible regenerative material candidates that prevent toxicity which may arise due to chemical crosslinking processes. Hydrogels can facilitate an extracellular matrix mimetic environment in relation to living cells for their migration and proliferation via their porous and water-abundant structures [1]. Chitosan-based in situ-forming pH- and thermosensitive injectable hydrogels are great candidates for minimally invasive tissue replacement and regeneration.

Successful tissue regeneration after injury, especially in highly vascularised tissues, such as bone, strongly depends on angiogenesis. Therefore, there is a tremendous need to develop novel pro-angiogenic strategies. One common technique to induce angiogenesis is the implantation of vein grafts in the arteries. However, this method requires a complex surgical method referred to as the arteriovenous loop technique. Other regenerative techniques are needed to combat the issue of ischemia and stimulate healing [2]. Another strategy in angiogenesis induction involves the use of pre-vascularised scaffolds produced by in vitro co-culturing of endothelial cells and mesenchymal stem cells. The major problems with the pre-vascularisation method may arise due to cell sources and the difficulties associated with the surgical binding of preformed vessels with native capillaries in the body [3,4]. The potential pro-angiogenic effects of different materials—bioceramics [4,5]; metal particles, including K-doped ZnO nanoparticles integrated into chitosan membranes [6]; sulphur-doped TiO nanoparticles integrated in chitosan/collagen membranes for use in ulcer- and burn-dressing materials [7]; and boric acid-loaded chitosan–collagen hydrogels [8]—have been investigated. In addition, growth factors (GFs), e.g., vascular endothelial growth factor, basic fibroblast growth factor, and transforming growth factor beta, have been widely reported and are among the strategies developed to stimulate angiogenesis since they can be used to monitor cell activities and the regeneration of tissues [9,10]. Despite their benefits, there are some obstacles to the delivery of growth factors, such as their short half-lives and instabilities which cause inefficient performances. It was reported that, as well as their high costs, using high concentrations of GFs may lead to serious adverse effects, e.g., inflammation and malformation or tumour-development in tissues [11,12].

To combat the instabilities of GFs, some researchers have reported the use of heparin or heparin-mimicking structures to bind GFs. Heparin is a natural proteoglycan composed of glycosaminoglycan chains bound to a protein; it is produced by mast cells in the body and is broken down into discrete molecules by endoglycosidases. Due to their anionic nature, heparins provide anchors for most GFs and protect them from fast degradation [13,14]. Heparin containing Konjac glucomannan hydrogels was reported to promote angiogenesis by macrophage activation [15]. Chitosan/deproteinated bone scaffolds coated with heparin were reported to have showed increased blood perfusion and angiogenesis [16]. In our group, heparin-loaded pro-angiogenic materials, including TEOF cross-linked poly(vinyl alcohol) (PVA)–chitosan hydrogels [17], PVA–polycaprolactone hydrogels for wound healing [18], and chitosan-derivative–PVA porous hydrogel membranes [19] have also been reported.

We earlier reported the development of heparin-conjugated chitosan-based composite pH- and thermosensitive injectable hydrogels neutralised by sodium bicarbonate (NaHCO_3_) which induced angiogenesis for bone regeneration [20]. Chitosan (CS)-matrixed biodegradable hydrogels were incorporated with bioactive substances—hydroxyapatite (HA) and heparin (Hep)—to achieve ionic and biomolecular anchorage between hydrogels and tissues. This was ensured by using the functionality of the carbonated hydroxyl groups in hydroxyapatite and anionic sulphate and the carboxyl groups in heparin to effect the binding of physiological angiogenic GFs to induce angiogenesis for eventual bone tissue regeneration. These hydrogels were obtained with unique injectability, gelation, and rheological features, and their pro-angiogenic potential was investigated by ex ovo chick chorioallantoic membrane (CAM) assays [20]. 

Despite very promising properties, hydrogels have limited mechanical durability because of their hydrophilic nature and high swelling potential. Therefore, various strategies are applied to enhance the mechanical strength of hydrogel matrixes. Chemical crosslinking methods by polymerisation are utilised to improve the mechanical properties of hydrogels. Thermosensitive and self-healing conductive hydrogels were reported based on the polymerisation of poly(N-isopropylacrylamide) (PNIPAAm) and PVA using borax as a crosslinker [21]. For the chemical crosslinking of chitosan-based hydrogels, the use of formaldehyde, epoxy, dialdehyde, glutaraldehyde [22], and genipin has been reported [23]. Although the chemical crosslinking methods can improve mechanical properties, they are associated with cytotoxicity due to crosslinker residues which require strict removal processes. Although less toxic crosslinkers, such as genipin, can be used, their usage in drug delivery applications is limited—in the case of genipin, due to unfavourable interactions with drugs [24]. Another approach to ensure crosslinking without crosslinkers is to increase the reactivity of polymers by modifying them by various chemical means, such as Schiff base and Michael addition reactions. However, these methods also create problems in terms of the purity and safety of the final products [24]. Therefore, the utilisation of chemical crosslinking processes might be quite detrimental.

Other strategies to enhance mechanical properties involve the manufacture of composites via the addition of inorganic particles or secondary polymeric phases. For instance, it has been reported that carboxymethyl cellulose matrix hydrogels strengthened by clay (laponite) nanoparticles which form semi-interpenetrated (IPN) elastomeric hydrogels [25]. Another study involved the use of carbon dots in alginate-based hydrogels to enhance their mechanical properties and simultaneously enable the binding of drugs for drug delivery applications for soft tissue engineering [26]. In addition, bioglass-reinforced injectable hydrogels produced by combination with natural polymers, such as gellan gum, alginate, chitosan, gelatin, and pectin, as well as synthetic polymers, e.g., PVA, polyethylene glycol (PEG), pluronic, and peptides, have been reported [27]. The integration of small amounts of eggshell-originated hydroxyapatite in alginate–silk fibroin to generate injectable composite hydrogels was also investigated for bone regeneration applications. Despite its high strength, the brittle structure of HA has been balanced by alginate—a gel that forms a biocompatible polymer which is mostly used as a thickening and stabilising agent—and fibroin, due to its intermolecular bindings [28].

CS-based injectable hydrogels can form physical hydrogels triggered by the dual effect of pH and temperature. The pH of CS solutions can be adjusted by different neutralising agents, such as β-glycerophosphate (β-GP) [29], Na_2_CO_3_ [30], and NaHCO_3_ [31]. Among these, β-GP has been studied most intensely. However, the high amount required for a neutral pH compromises cytocompatibility and cost-effectiveness, and mechanically weak hydrogels were obtained with its use. Therefore, utilisation of a weak base, such as NaHCO_3_ with β-GP, has been reported to improve biocompatibility and mechanical properties simultaneously [32].

In our initial CS-based hydrogels (CS/HA/Hep), in which NaHCO_3_ was used as a neutralising pH, homogeneous injectable solutions were obtained by the gradual addition of NaHCO_3_. However, low-pH solutions (6.2–6.3 at 4 °C) were obtained and the hydrogels had limited mechanical stabilities [20]. Therefore, in this current study, we investigated the use of glycerol in CS solution media as a stabilising agent to maintain solution homogeneity at higher pH levels adjusted by NaHCO_3_. Glycerol is a transparent, viscous, and sweet polyol which has a density of 1.261 g/mL and melting and boiling points of 18.2 °C and 290 °C, respectively. Glycerol can be used as a stabilising and moisturising additive. Being non-toxic and low-cost, glycerol is used in a wide range of industrial applications, including food, cosmetics, pharmaceuticals, biofuels, biosensors, and biodiagnostics [33]. In this study, by means of the multifunctionality of glycerol, pH increments towards physiological levels with preserved solution homogeneity, and further pH-induced gelation, stronger, interpenetrating hydrogels were produced. Furthermore, for pre-sterile hydrogel preparation, glycerol has served as a protective agent for CS dispersions sterilised by heat. To the best of our knowledge, the use of readily available glycerol in a pH- and temperature-sensitive CS-based injectable hydrogel system neutralised by NaHCO_3_ has not been investigated before. The synergetic effects of glycerol addition and pH factors on the gelation, microstructural, and mechanical properties of CS/HA/Hep hydrogels were thoroughly examined.

As reported, hydroxyl moieties in glycerol cover CS chains and protect the polymers from degradation, which can be caused by heat sterilisation [34,35]. Sorlier et al. (2001) [36] reported that the pKa of polymers can change due to electrostatic interactions in CS solutions. Adding glycerol to CS solutions leads to hydrophobic interactions due to CS’s hydroxyl groups. This results in less interaction of the functional groups in CS. Thus, glycerol allowed further pH increments during neutralisation with NaHCO_3_ which could have been be due to an increase in the pKa points of the solutions. However, this did not impair solution homogeneity, and injectable solutions without precipitation were acquired with high pH values. Following the evaporation of liquid media during gelation, glycerol led to inter- and intra-hydrogen bonding with chitosan, and increased salt concentration resulted in further pH- and temperature-induced crosslinking in hydrogels at average body temperature (37 °C). Delmar and Bianco-Peled, (2015) [37] also reported the significant impact of small pH changes on the gelation of CS hydrogels when CS was cross-linked with genipin. Gelation time was decreased by increasing pH, and swelling and other physical properties were also affected by pH.

This is an elaborated study which examines the synergetic effect of glycerol and pH factors on diverse properties of CS-based, dual pH- and temperature-sensitive injectable hydrogels (CS/HA/Hep). Gelation properties and microstructural and mechanical changes were analysed in detail. Since pH is the main driving force in gelation, incipient gelation time was decreased upon increasing pH with additional NaHCO_3_ salt, and further crosslinking in hydrogel networks occurred upon heating at 37 °C. The glycerol-modified hydrogels produced in high-pH solutions (6.4–6.5 at 4 °C and 7.0–7.2 at 20 °C) started gelation in only 2–3 min at 37 °C. Hydrogels obtained in high-pH solutions showed hierarchical porosity and stronger interlocking cross-linked structures. Anionic Hep molecules positioned at the top of the hydrogels formed a bioactive surface. In angiogenesis tests using the CAM assay, hydrogel solution drops had a proper density to remain at the implantation site without any leakage on chick embryo membranes. CS/HA/Hep hydrogels with a Hep concentration of 33 µg/mL showed significantly higher pro-angiogenic responses than simple CS hydrogels.

## 2. Results

### 2.1. Injectability and Gelation Properties

The pre-sterile preparation of hydrogel solutions with glycerol added to the mixtures led to an increase in the final pH value above 6.3 by maintaining solution homogeneity. Further neutralisation with NaHCO_3_ at 4 °C up to a pH range of 6.7–6.8 did not cause precipitation initially; however, phase separation occurred after the synthesis. Therefore, the final pH of solutions was kept at a maximum of approximately 6.55 at 4 °C. These solutions were stable for 4 days in a refrigerator (at 4 °C) without phase separation. At 20 °C, composite hydrogel solutions had a pH between 7 and 7.2 (Table 1).

**Figure 1:**
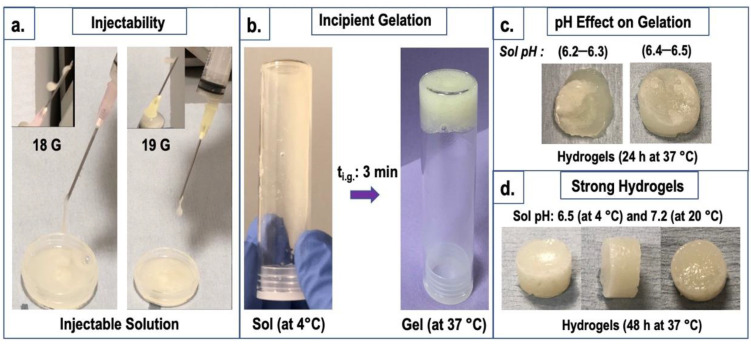
Diagram demonstrating the different properties of composite hydrogels, S0: (**a**) injectability of a solution (pH: 6.4–6.5, at 4 °C) upon synthesis through 18-gauge and 19-gauge needle-coupled syringes (5 mL); (**b**) incipient gelation time (t_i.g._) of a solution (pH 6.4–6.5) in a test tube at 37 °C determined as 3 min; and (**c**) pH effect on gelation: weaker, non-stable hydrogels were obtained with a final solution pH of 6.2–6.3, whereas higher pH solutions (6.4–6.5) resulted in properly formed hydrogels after 24 h of incubation at 37 °C; (**d**) strong hydrogels: the appearance of hydrogels after 48 h of incubation at 37 °C obtained from high-pH solutions (pH: 6.5, at 4 °C; and 7.2, at 20 °C).

After the synthesis, the injection of hydrogel solutions against air through 21-gauge and thicker-sized needles was very convenient. Solutions showed a constant flow through an 18-gauge needle and fast dropwise flow through a 19-gauge needle attached to a 5 mL syringe (Figure 1a). However, upon storage for 24 h at 4 °C, due to increased solution viscosities, the injectability capacity decreased. Following 10 days of storage, injection through 18-gauge needle was possible with a moderate pressure.

Gelation started at 5 min in the sole CS composition (CI), whereas the gelation of the composite hydrogel solutions (S0 and SI) initiated in just 3 min (Figure 1b). Further incubation (at 37 °C) of hydrogel solutions with a pH below 6.4 for 24 h resulted in weak and not-properly-formed hydrogels. However, in solutions with a higher pH range (above 6.4), properly formed, stable, strong hydrogels were acquired after 24 h (at 37 °C) (Figure 1c). Before the analyses were performed, the hydrogels were incubated for 48 h at 37 °C. The images of much stronger hydrogels obtained with higher pHs (6.5 at 4 °C and 7.2 at 20 °C) are shown in Figure 1d.

### 2.2. Morphology by SEM

**Figure 2:**
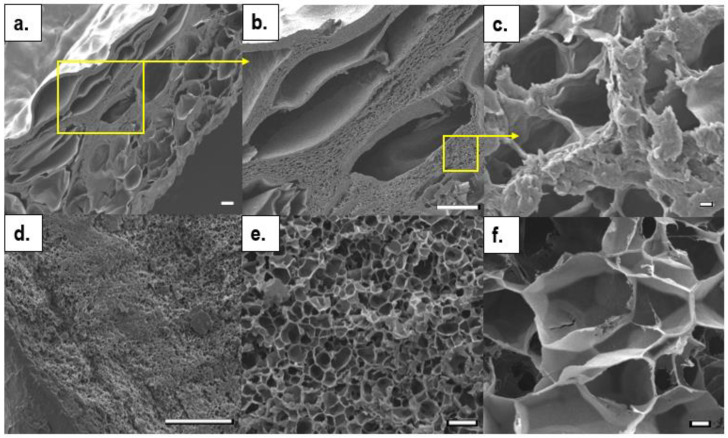
The comparative microstructure SEM images of half cross-sectioned hydrogels (CI) obtained from solutions with two different pH ranges: (**a**–**c**) 6.4–6.5; and (**d**–**f**) 6.2–6.3. (Scale bars: (**a**,**b**,**d**): 100 µm; (**e**): 10 µm; (**c**) and (**f**): 1 µm.).

The microstructural properties of freeze-dried hydrogels were examined by SEM. The results showed that all hydrogels presented a morphology with a smooth, flat bottom surface and a rougher top surface with a small number of pores, most of which were at cross-section regions. The small increments in pH from 6.20–6.35 to 6.40–6.55 had significant impacts on the final structural morphologies of the hydrogels. The lower pH group had a more homogeneous pore structure. However, the very small pore sizes might have been due to excess drying of the analysed hydrogels, which became thin film specimens (Figure 2d–f).

**Figure 3:**
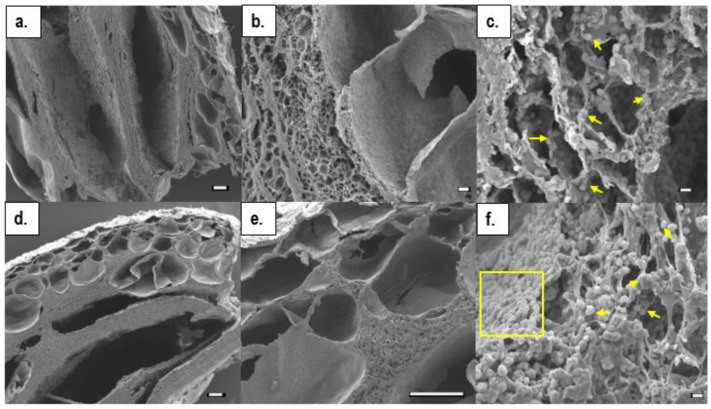
The SEM images of half cross-sectioned composite hydrogels obtained from solutions with pHs of 6.4–6.55: (**a**–**c**) CII; and (**d**–**f**) SI. (Scale bars: (**a**,**d**,**e**): 100 µm; (**b**): 10 µm; (**c**) and (**f**): 1 µm.).

At higher pHs, however, hierarchical, interpenetrated pore morphologies were presented (Figure 2a,b); large macropores had mean pore sizes of 200 ± 10 µm and minimum and maximum pore diameters of 30 µm and 500 µm, respectively. The separating pore walls with small, dense microporosities were measured separately. In this region, the mean, minimum, and maximum pore diameters were 3 ± 1.5 µm, 0.5 µm, and 6.5 µm, respectively (Figure 2c). SEM microstructural images were taken of the composite hydrogels, CII and SI, produced at high pH solutions (6.4–6.55), and are shown in Figure 3. These also possessed a range of pore sizes, as well as thick pore walls with dense micropores. Hydroxyapatite crystals were dispersed on the surfaces of pores at cross-sectional regions.

The mean pore sizes at the cross-section regions of CII and SI in the sample micrographs were 400 µm and 240 µm, respectively, while maximum pore sizes reached 1.5 mm in both. At separating micropores, mean pore sizes ranged between 3–3.5 µm and 8.5 µm.

### 2.3. Chemical Analyses by FT-IR

**Figure 4:**
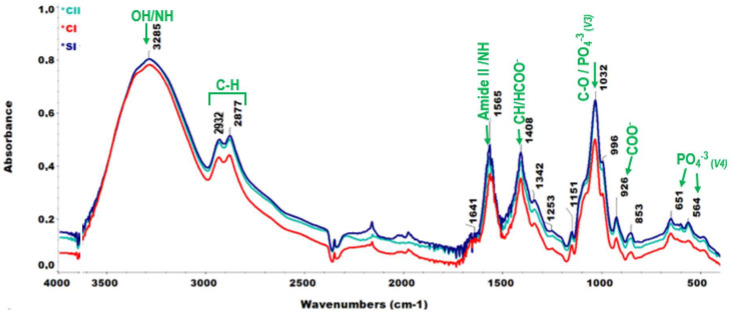
ATR spectra of hydrogels with diverse formulations: CI, CII, and SI.

The chemical structures of hydrogels were determined by FT-IR. The ATR spectra of hydrogels at different formulations are presented in Figure 4. The spectra of the simple CS hydrogel (CI) had slightly less intense peaks than the composite hydrogels (CII and SI) with crystalline HA. The broadest peak in the ATR spectra of all hydrogels at a wavelength of 3285 cm^−1^ was referred to an overlap in the stretching frequencies of O–H and N–H bonding [38]. This broad single peak showed a less crystalline nature of the hydrogels and the dominance of hydroxyl groups contributed by glycerol positioned in the vicinity of 3280 cm^−1^ [39].

C–H stretching vibrations were observed as shoulders of the broadest peak and these were designated at 2877 cm^−1^ and 2932 cm^−1^. The sharp peak located at 1565 cm^−1^ was attributed to amide II or N-H bending; specifically, this might be associated with symmetric deformation of NH_3_^+^ cations in CS. The adjacent sharp peak at 1408 cm^−1^ corresponded to the bending mode of C-H vibrations or, particularly, carboxylic acid (HCOO^-^) bonds [38,40]. A tiny peak seen at 1641 cm^−1^ was a result of amide I or C=O vibrations. The shoulder raised at 1151 cm^−1^ was determined as having been due to C-O bond stretching [19]. The peak at 926 cm^−1^ corresponded to symmetrical stretching bonds of COO^-^ due to acetate molecules [41].

The steep peak with a high intensity at 1032 cm^−1^ corresponded to C-O cyclical stretches in CS [42,43] and stretching (asymmetric) of PO_4_^−3^ (ν_3_) molecules in crystalline HA particles [44] present in composite hydrogels (CII and SI). Other phosphate bonds in composite hydrogels with HA led to stronger peaks at 651 cm^−1^ and 564 cm^−1^, which corresponded to ν_4_ bending vibrations of PO_4_^−3^ ions [45]. O-C-O, ν_2_ bending moieties of CO_3_^−2^ molecules were located at 857 cm^−1^ [46].

### 2.4. Compression Strength Tests

**Figure 5:**
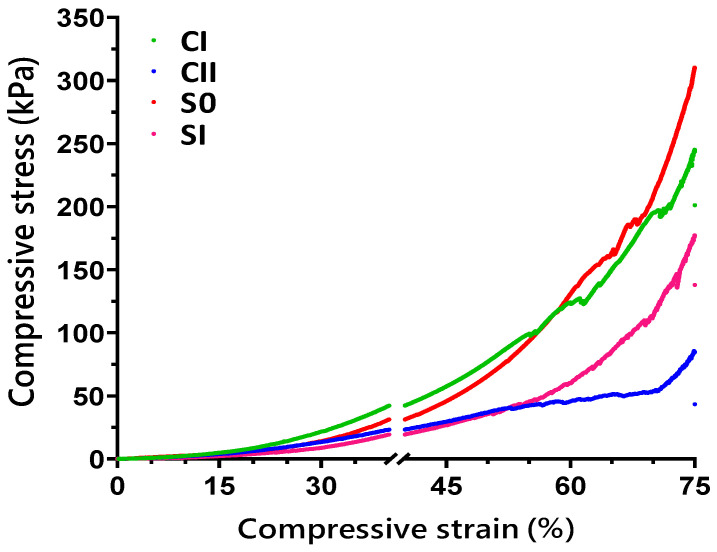
The curves of mean compressive stress versus strain (maximum 75%) obtained for hydrogels (solution pH of 6.4–6.5) in diverse formulations: CI, CII, S0, and SI.

Mechanical tests on hydrogels were performed via compressive strength measurements. The compression strength of lower-pH solution-hydrogels performed up to 40% of strain and showed approximately 15 kPa of maximum compression stress for all compositions (data not shown). The compressive stress–strain curves of higher-pH hydrogels with different compositions up to 75% of strain are shown in Figure 5.

A progressive deformation was detected in hydrogels which might have been associated with their entangled hierarchical and porous microstructures dissipating energy due to applied force. Base CS (CI) hydrogels had initial deformations at around 56% of elongation, while this level was raised to a strain of approximately 69% in composite SI (CHH120) hydrogels. Hydrogel compositions were not exposed to total breakage at 75% of strain (end of tests). The composite S0 hydrogels (CHH33) were found to sustain the maximum compressive stress of 310 kPa and had a Young’s modulus of 1.5 MPa at 75% strain.

### 2.5. Heparin (Drug) Detection and Release 

**Figure 6:**
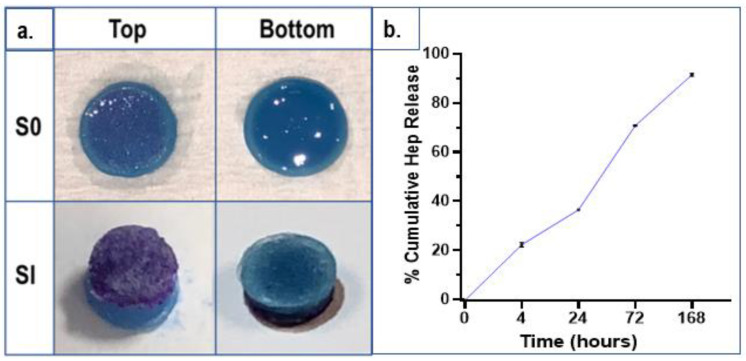
(**a**) Local Hep detection by toluidine blue-staining of composite hydrogels (7 mm diameter, 5 mm thickness) obtained from the high pH solutions (6.4–6.55 at 4 °C): S0 with a Hep concentration of 33 µg/mL and SI with a Hep concentration of 120 µg/mL (below) (top and bottom images). (**b**) The percentage cumulative drug release profile of Hep eluted from hydrogels obtained from the low-pH solutions (6.2–6.35 at 4 °C) (S0: 33 µg/mL Hep) into de-ionised ultrapure water (D.H_2_O) incubated at 37 °C, at different time periods. Hep amount was counted by the change in the absorbance values of non-complexed TB extract solutions and correlated with the standard curve. (Three sample replicates were analysed and the data are shown as standard deviations.).

The presence of Hep in cross-linked hydrogels was determined by toluidine blue (TB) staining assay. In composite hydrogels with Hep, the regions containing Hep were stained a purple colour while other regions were blue. Figure 6a shows the stains of two different hydrogel compositions: S0 (CHH33) and SI (CHH120), top and bottom surfaces. Hydrogels (low-pH group) with low Hep concentrations (S0) were analysed by TB assay and a cumulative drug release profile is presented in Figure 6b.

As seen in the photographs in Figure 6a, the bottom surfaces of hydrogels are blue, indicating the absence of Hep, while the top surfaces of these compositions are stained a purple colour which was more intense in the SI sample with a higher Hep concentration than S0. These results revealed the formation of a functional Hep located on the surface which can contribute to the bioactivity. Regarding the drug release profiles of S0 hydrogels (low-pH specimens) (Figure 6b), after 4 h, approximately 20% of Hep was released and this reached around 40% in 24 h. After 3 days, 70% of Hep was released and nearly 8% of Hep remained after the end of the test on the 7th day.

### 2.6. Angiogenesis Analyses by Ex ovo CAM Assay

**Figure 7:**
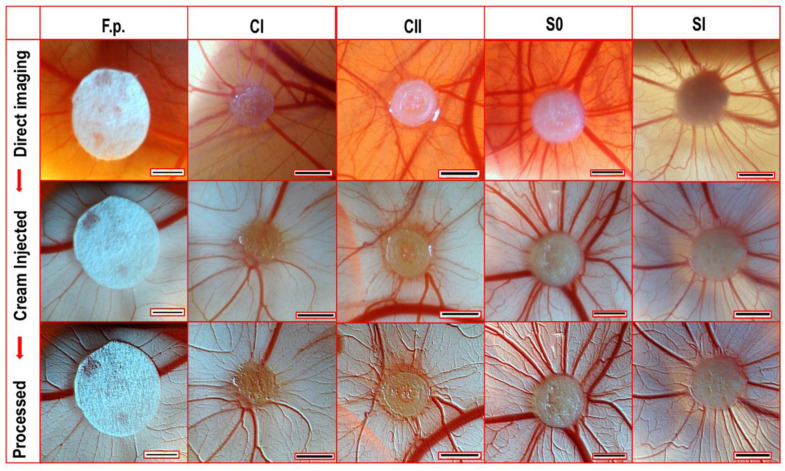
The comparative CAM images taken at day 14 of the assay. (All scale bars are 2 mm.) Test groups: Filter papers treated with PBS (F.p.s) as controls; CI and CII hydrogels without Hep; and S0 (33 µg/mL Hep) and SI (120 µg/mL Hep) with Hep. The images in rows from top to bottom show, respectively: direct imaging of CAM, imaging after white-cream injection underneath the CAM (as a contrast for red blood vessels), and the processed (north-east shadow) image versions of the middle images (in ImageJ Software). (The sizes of the filter papers (Ø: 6 mm) were equal to the sizes of the hydrogels at implantation day (day 7) but the hydrogels shrank to smaller sizes during incubation at 37 °C.).

The effects of hydrogel samples on angiogenesis were tested by CAM assay performed using chick embryos. CAM images were taken 6 days after the implantation of hydrogels. Filter papers (F.p.s) treated with phosphate-buffered saline (PBS) were used as controls (Figure 7). Although the sizes of F.p.s and hydrogels were equal on the day of implantation (Ø: 6 mm), hydrogels shrank to almost one-third of their original sizes at day 14. There were small numbers of blood vessels around F.p.s, but they showed almost no interaction with F.p.s. However, blood vessel infiltration into hydrogels was detected well. The extent of neovascularisation was least with the simple CS hydrogels (CI). The addition of hydroxyapatite contributed a slight increase in the number of microvessels in the CII specimens. The greatest microvascularisation effect was observed with hydrogels containing Hep. The S0 hydrogel with the smallest concentration of Hep (33 µg/mL) developed the most mature and the greatest number of blood vessels.

**Figure 8:**
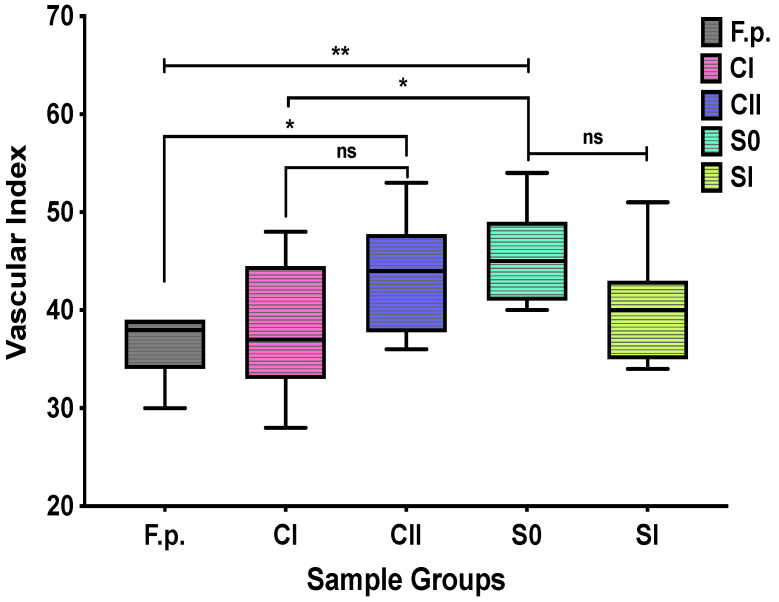
CAM image (at day 14) analyses represented as vascular index counts with means ± SDs (9–11 images from each sample group *(n = 1)*). Test specimens: filter papers treated with PBS (F.p.s) as controls; CI and CII hydrogels without Hep; S0 (33 µg/mL Hep) and SI (120 µg/mL Hep) with Hep. The *p*-values calculated based on one-way ANOVAs were: CI and S0 (*): 0.0298; F.p.s and CII (*): 0.0287; F.p.s and S0 (**): 0.0026; S0 and SI (ns): 0.2902.

The quantitative results of the angiogenesis analyses are shown as *vascular index* in the sample groups in Figure 8. The composite S0 hydrogel (with 33 µg/mL Hep) had the maximum vascular index count of 45.7 ± 4.7. The vascularity of the CII and SI samples showed similar behaviour. The comparison values for S0 and SI samples were not statistically significantly different (*p* = 0.2902). However, vascular index of S0 hydrogels showed statistical significance upon CI hydrogels (*p* = 0.0298) and F.p.s (*p* = 0.0026).

## 3. Discussion

The modified pre-sterile synthesis significantly altered the microstructural and mechanical properties of the CS/HA/Hep hydrogels. The glycerol added in CS–water dispersions prior to autoclaving served as a heat-protective agent. Furthermore, due to the effect of glycerol, the pH of injectable solutions could be increased to a higher range by the addition of NaHCO_3_, as solution homogeneity was maintained. The modified injectable solutions showed thicker, elastic flow properties which may prevent leakages and be advantageous for solutions injected to target tissue defects. During the synthesis, the injectability of hydrogels was evaluated using 21-gauge needles according to a procedure reported previously [20]. However, upon storage at 4 °C for 24 h, solutions became more viscous, and 18-gauge needles were more suitable for injection. Interestingly, once solutions were warmed to room temperature, they became more fluid, but formed strong hydrogels at 37 °C. This could be related to the alteration of freezing points of glycerol based on its concentration at different temperatures [47]. Therefore, the long-term optimal storage conditions for these solutions is a matter that requires further research. In addition, heat sterilisation may also have some detrimental effects on CS. The sterilisation of degradable, injectable polymer solutions remains a challenge. As a result of an extensive literature search regarding sterilisation techniques [48], we decided to use heat-sterilisation of CS in aqueous media by adding glycerol for heat protection.

Two different pH ranges of final injectable solutions (at 4 °C) were compared in this study: a low pH range of 6.20–6.35 for initial hydrogel solutions; and a high pH range of 6.40–6.55. The pH values of the high-pH solutions at 20 °C were between 7 and 7.2, which are more compatible with the physiological pH ranges of body fluids [29]. To provide suitable injectability and physiological pH in the final solutions, following synthesis at 4 °C, warming solutions to around 20 °C might be more suitable for administering them into targeted tissue defects. This study showed the collaborative effects of glycerol additive and (leading) small pH increments, which resulted in enormous changes in gelation speed, and the microstructural and mechanical features of hydrogels. In comparison with low-pH hydrogel solutions, their high-pH counterparts apparently triggered gelation, since incipient gelation time was reduced from 5–10 min to 2–3 min. Hep addition also decreased gelation time from 5 min to 2–3 min, which could have been due to the effect of affinity bonding between CS and Hep in a polycomplex system. Chitosan contains a positive charge on its surface, heparin a negative charge; these charges on the surfaces are potentially responsible for faster gelation. The hydrogels obtained from low-pH solutions showed uniform porous microstructures (as initial hydrogels). In their high-pH counterparts, however, hierarchical porosities with macropores 50–500 μm in size separated by dense micropore walls (1–10 μm) were detected. The effects of pH on gelation time and morphology were inevitable since the sol–gel transition of CS is mainly driven by pH due to the release of carbon dioxide during the neutralisation reaction between protonated amino groups of CS in acidic media and carbonic acid groups from sodium bicarbonate salt [31]. At this point, glycerol served to increase the pKa of polymers that stabilised the CS solution, allowing a further pH increment by maintaining the homogeneous, injectable nature of the solution. Further thermosensitive crosslinking of CS occurred with an increase in temperature which involved the evaporation of liquid media and the formation of hydrophobic junctions. The flower-like porous HA crystals (own synthesis) were mostly dispersed on the pores at the cross-section region. Hep adopted a coating-like structure on rough HA and salt crystals. Heparinised surfaces seem softer but anionic surface properties are considered attractive for the attachment of biological molecules, enhancing bioactivity, as was confirmed later by SBF tests (unpublished data).

Hydrogels obtained at elevated pHs showed a big enhancement in compressive strength and elastic modulus. Composite hydrogels (CS/HA/Hep) exhibited greater Young’s moduli than notified values for articular cartilage (0.45–0.80 MPa) [49,50]. The maximum stress at 75% of elongation was 310 kPa and Young’s modulus was 1.5 MPa for S0 (CS/HA/Hep) hydrogels. Hydrogels showed a progressive deformity which might be associated with hierarchical interpenetrating hydrogel networks, with ranged porosity dissipating compression force and delaying failures. Compressive stress measurements for hydrogels at initial deformation levels were 51–172 kPa, which are considered sufficient to ensure the differentiation of osteogenic stem cells (mentioned as 45–49 kPa) [51,52]. Hydrophobic and hydrophilic sites emerged as a result of glycerol–water media interacting with CS, leading to inter- and intra-bonding in hydrogel networks [53,54]. As reported, glycerol-treated chitosan–gelatine cross-linked hydrogels exhibited enhanced hydrogel elasticity and resilience [55]. Ahmad et al. 2012 [56] also noted that, by enhancing flexibility, plasticizers provide stress relaxation features, reducing deformities and strengthening structures. The addition of PEG to alginate-based hydrogels resulted in biomimetic and tuneable hydrogels with stress relaxation properties that led to enhanced biocompatibility with fibroblasts and osteogenic stimulation [57].

In this study, Hep in hydrogel networks was located on the top layers of hydrogels, forming bioactive surfaces. Hep release studies with low-pH hydrogels showed that Hep has a progressive delivery profile and a small amount of Hep (8%) remained in hydrogels after 7 days of release. Lu et al. 2007 [58] investigated Hep delivery from silk fibroin–collagen scaffolds using the same TB assay. The results showed that around 40% of Hep was released during 24 h; the remaining amount had a more prolonged release of up to 8 days. He et al. 2010 [59] also reported a more sustainable release of Hep with a chitosan–heparin scaffold and films. It is supposed that the higher-pH hydrogels in this study could provide much more sustained releases of Hep due to inter- and intra-hydrogen bonding creating stronger hydrogel networks. In the CAM studies, the densities of the modified injectable solutions were appropriate to remain circular at the injection sites on the CAMs without any leakages. The most profound pro-angiogenic effects, with developed microvascularity, blood vessel infiltration, and maturation, were seen with composite hydrogels integrated with HA crystals and a small quantity of Hep (33 µg/mL). This composition (S0) had a significantly higher vascular index count (45.7 ± 4.7) than those of the CI sole CS (p = 0.0298) hydrogels and F.p.s (p = 0.0026). HA particles slightly increased microvascularity compared to base–CS hydrogels. This can be explained by the influences of bioceramics on the angiogenesis addressed by the researchers [4,5].

The hierarchical macro- and microporosities of hydrogels obtained with higher pHs seem very beneficial in terms of their biological responses. It has been stated that the presence of 10–20% of micropores (<20 μm) as well as macroporosity (>50 μm) increases permeability in HA scaffolds. Thus, microporosity has triggered rapid bone ingrowth due to an increase in permeability ensuring the transport of vital agents, e.g., oxygen, nutrients, and cells, leading to bioactivity, osteointegration, and angiogenesis [60,61]. In our study, due to enhanced permeability in hierarchical porous hydrogels, the release of Hep is expected to occur in a more sustained manner, and blood infiltration and transportation are expected to be enhanced, both of which contribute to angiogenesis. Therefore, this study shows the substantial effects of hydrogel chemistries involving pH increments and glycerol additives which led to a unique hierarchical and strong hydrogel morphology. Hence, these composite hydrogels, with efficient drug delivery capacity and bioactive and proangiogenic performances, might be great candidates for advanced, minimally invasive bone tissue engineering and drug delivery applications. 

## 4. Materials and Methods 

### 4.1. Materials

For the production of HA particles, the following chemicals were acquired: calcium nitrate tetra-hydrate (Ca(NO_3_)_2_·4H_2_O) (Acros Organics, Geel, Belgium); di-ammonium hydrogen phosphate ((NH_4_)_2_HPO_4_) and ethanol (≥99.8%, AnalaR NORMAPUR^®^) (VWR-Prolabo Chemicals, Lutterworth, UK); and ammonium hydroxide (NH_4_OH) (35%, Thermo Fisher Scientific, Leicestershire, UK). The reagents used for hydrogel manufacture were as follows: Chitosan (100–300 kDa, Deacetylation degree: ≥90%), glacial acetic acid, glycerol (99%) (ACROS Organics^™^, Thermo Fisher Scientific, Geel, Belgium); sodium bicarbonate (NaHCO_3_) (Fluka^®^, Sigma Aldrich, Morris Plains, NJ, USA); and sodium bovine heparin (Injectable grade, 156 IU/mg) (a kind present from Extrasul (Ext. An. Veg. LTDA, Sao Paulo, Brazil)). For the analyses, n-hexane (95+%), 1N-HCl and Phosphate-Buffered Saline (PBS) (Thermo Fisher Scientific^™^, ACROS Organics^™^, Geel, Belgium) were purchased. De-ionised ultrapure (Type-I) water (D.H_2_O) (Veolia, PURELAB^®^ Chorus, 18.2 MΩ.cm, Wycombe, UK) was used in all studies.

### 4.2. Production of Synthetic Hydroxyapatite by the Sol–Gel Method

Synthetic hydroxyapatite (HA) particles were manufactured by utilising a sol–gel synthesis route adapted from the literature [62]. For a stoichiometric HA reaction, 0.5M Ca(NO_3_)_2_.4H_2_O and 0.3M of (NH_4_)_2_HPO_4_ were dissolved in equal volumes of (500 mL) ethanol and D.H_2_O, respectively. Subsequently, the pH of these solutions was fixed at 10.5 using NH_4_OH solution. Then, the phosphate precursor solution was mixed dropwise with Ca(NO_3_)_2_.4H_2_O precursor at 85 °C by constant stirring. The reaction was maintained for 4 h by keeping the pH at 10 (at 85 °C). HA precipitates were then acquired by filtration and sufficient washing to achieve a solution with neutral pH. HA powders were acquired upon drying at 200 °C and sieving under 150 µm.

### 4.3. Fabrication of Injectable CS/HA/Hep Hydrogels

Hydrogels were synthesised through a pre-sterile preparation technique in a sterile fume hood. Solutions of Hep and NaHCO_3_ were produced by dissolving Hep and NaHCO_3_ in D.H_2_O and then passing the solutions through filters (0.22 µm polyethersulfone (PES)) for sterilisation. CS powders (1 g) as dispersions in 20 mL of D.H_2_O with glycerol added (5% wt/v) were sterilised by autoclaving. Then, sterile CS dispersions were dissolved in glacial acetic acid (0.5 M of solution). HA was autoclaved as a powder, then 0.1 g of sterile HA was blended into a CS solution by constant stirring overnight. Sterile Hep solution (5 mL) was added dropwise into the CS/HA mixture. Subsequently, the CS/HA/Hep solution was chilled for 15 min at 4 °C and neutralised in an ice bath by dropwise addition of 0.48 M of NaHCO_3_ (at 4 °C) solution. Hydrogels were manufactured by incubation of solutions at 37 °C. Hydrogel samples without Hep with sole CS and CS/HA compositions were also synthesised similarly by neutralisation and were referred to as CI and CII, respectively. Hep-containing composite CS/HA/Hep hydrogels were denoted S0 and SI, with 33 µg/mL and 120 µg/ mL of Hep in their respective final solutions.

### 4.4. Morphology Analyses by SEM

Before SEM imaging, hydrogels were exposed to a freeze-drying (−20 °C) process and gold-coating was applied (5 nm). Then, hydrogels were imaged (top, bottom, and vertically half-sections) at an accelerating voltage of 5 kV, using a Schottky FE-SEM instrument (JEOL JSM-7800F, Tokyo, Japan).

### 4.5. Chemical Analyses by FT-IR 

Hydrogels were chemically characterised by the Attenuated Total Reflectance (ATR) technique in an FT-IR spectrophotometer (Thermo Nicolet^™^ iS50, Thermo Fisher Scientific, Inc., Madison, WI, USA) with a detector, DTGS ATR, and a and a beam splitter, KBr. Analyses were performed by working in the mid-infrared region (400–4000 cm^−1^) with an aperture of 150 and a resolution of 16 cm^−1^. Acquisition of spectra occurred after 128 scans, and these were analysed after automatic baseline correction with OMNIC^TM^ software (Version 9.5.9, Thermo Fisher Scientific, Inc., Madison, WI, USA).

### 4.6. Local Heparin Detection by Toluidine Blue Staining

Toluidine Blue (TB) powder was dissolved in 0.01 N solution of HCl (0.005 % (wt/v)) containing 0.2% NaCl [63]. Hydrogels were stained by immersion in TB solution for 30 min and unbound stains were washed thrice with D.H_2_O. Then, stained hydrogels were imaged with a mobile phone to indicate the local presence of Hep in hydrogel networks.

### 4.7. Drug Release Studies by Toluidine Blue Assay

TB assays were conducted as reported [58,64]. First, Hep standard solutions were produced in D.H_2_O at known concentrations (0, 20, 40, and 60 μg/mL). Each standard solution (1 mL) was blended with TB solution (1.5 mL) and rested for 30 min. Then, these blends were sufficiently swirled to form TB–Hep complexes by the addition of hexane (1.5 mL). The non-reacted TB solutions under the complexes were collected. The optical density measurements of these liquids were taken at 630 nm. These data were plotted against known Hep concentrations to obtain a standard curve. To determine the drug release of hydrogels, cylindrical hydrogel specimens were prepared in triplicate, and after weighing (~0.1 g) they were incubated in D.H_2_O (10 mL) at 37 °C. At test intervals (4, 24, 72, and 168 h), TB–Hep complexes were formed by the reaction of Hep-released solutions (1 mL) and TB, adding hexane as aforementioned. The correlations between the optical density values of the non-reactive TB liquid phases and the standard curve equations gave the amounts of released Hep.

### 4.8. Mechanical Analyses by Compression Strength Tests

For compression tests, hydrogels (diameter: 16 mm; height: 8 mm) were produced from liquid blend forms (2 mL) in test tubes by incubation at 37 °C for 48 h. Analyses were conducted in universal testing machine (Instron^®^ 3345, Norwood, MA, USA) in conjunction with a 0.5 kN load cell. Hydrogels were exposed to a compression of up to 75% of elongation at a momentum of 1 mm/min.

### 4.9. Angiogenesis Analyses by CAM (Ex Ovo) Assay 

A chick chorioallantoic membrane (CAM) assay was conducted by applying an ex-ovo method for in situ-formed injectable hydrogels as we reported earlier [20]. Briefly, fertilized chick eggs (Henry Stewart & Co., Ltd., Louth, UK), purified of dirt and germs (using 20% ethanol), were incubated horizontally (for 3 days) in moisturised, rotational egg incubators (at 37.5 °C). At development day 3, eggshells were removed and egg contents were taken out (the chick embryos were on top) utilising an ex ovo, sterile method. At day 7, hydrogel solutions (100 µL) were dropped onto CAM tissues of embryos using a micro-syringe and turned into hydrogels after a few minutes at 37 °C. Microvascularity in contact with hydrogels was photographed at days 10 and 14 with a portable microscope (Maplin, (×)400). The number of blood vessels attached to hydrogels (9 to 11 specimens for each sample group) was calculated by means of the ‘vascular index’ technique [65] using ImageJ^®^ software (Version 1.52 k, National Institutes of Health, Bethesda, MD, USA).

### 4.10. Statistical Analyses

Statistics analyses of the collected data were conducted using GraphPad Prism software (Version 7.0, San Diego, CA, USA). Tukey’s multiple comparison test (one-way ANOVA) was employed for statistical analysis of the CAM assay results. A *p*-value ≤ 0.05 was considered statistically significant.

## 5. Conclusions

The functionalisation of CS/HA/Hep hydrogels by glycerol in a pre-sterile synthesis led to further pH increments in homogeneous injectable solutions. Freshly prepared hydrogel solutions were conveniently injectable using 21-gauge needles. Although heat sterilisation of CS in hydroalcoholic media did not compromise gelation, it caused an increase in the viscosities of solutions during storage, though solutions were still injectable with 18-gauge needles. An increase in pH ranges from 6.20–6.35 to 6.40–6.55 reduced the incipient gelation times of the solutions by 2 to 3 min. Hydrogel networks had a flat bone-like morphology having hierarchical porosity which consist of large pores at the cross-sections (50–500 µm) with intersecting micropore (1–10 µm) walls. Flower-like porous HA crystals covered the porous surfaces thoroughly. As a result of the combinational effects of glycerol and pH, the mechanical strengths of CS/HA/Hep hydrogels have been significantly improved. As confirmed by the SEM and TB analyses, Hep coated the top surfaces of hydrogels, functionalising bioactive and pro-angiogenic features. Owing to the optimised composition (S0 with 33 µg/mL Hep) facilitating the best induction of angiogenesis, CS/HA/Hep hydrogels functionalised by glycerol have great potential for use in enhanced, minimally invasive, vascularised tissue regeneration and drug delivery systems.

## Figures and Tables

**Table 1:** The pH measurements at homogeneous solutions after synthesis, at 4 °C and at 20 °C, and the incipient gelation time (ti.g.) in a test tube (at 37 °C), with sample codes given for different compositions of hydrogels. CI (C) and CII (CH) are sole CS hydrogels and CS/HA composite hydrogels, respectively. CS-, HA-, and Hep-containing hydrogels are coded as S0 (CHH33) and SI (CHH120); these contained 33 µg/mL and 120 µg/mL of Hep, respectively.

Samples	Solution pH		* (t_i.g._, min)
	4 °C	20 °C	
CI	6.43	6.78	5
CII	6.42	7.00	4
S0	6.54	7.16	3
SI	6.46	7.00	3

* t_i.g._: Incipient gelation time (minutes).

## Data Availability

Not applicable.

## Abbreviations

ATR	Attenuated Total Reflectance
CAM	Chick Chorioallantoic Membrane
CS	Chitosan
ECM	Extra Cellular Matrix
FE-SEM	Field Emission-Scanning Electron Microscopy
FT-IR	Fourier Transform-Infrared Spectroscopy
GFs	Growth Factors
HA	Hydroxyapatite
Hep	Heparin
NaHCO_3_	Sodium Bicarbonate
PBS	Phosphate Buffer Saline
PEG	Polyethylene Glycol
SEM	Scanning Electron Microscopy
